# The impact of exercise interventions before, during, and following neoadjuvant therapies for locally advanced rectal cancer: a critical review

**DOI:** 10.2340/1651-226X.2026.44930

**Published:** 2026-05-20

**Authors:** John Saxton, Chizitara Amadi, Victoria Brown, Mohan Hingorani, Rajarshi Roy

**Affiliations:** a School of Sport Health, Exercise, and Rehabilitation Science (SHERS), University of Hull, Hull, England; b School of Sport Health, Exercise, and Rehabilitation Science (SHERS), Post Graduate Office, School of Sport Health and Exercise Building, University of Hull, Hull, England; c Specialist Cancer and Support Services Care Group, Hull University Teaching Hospitals NHS Trust, Hull, England; d Clinical Oncology, Hull University Teaching Hospitals NHS Trust, Cottingham, England

**Keywords:** exercise, physical activity, neoadjuvant chemoradiotherapy, rectal cancer, locally advanced rectal cancer, feasibility

## Abstract

**Background and purpose:**

Neoadjuvant chemoradiotherapy (NACRT) and radiotherapy (NART) are standard treatments for locally advanced rectal cancer (LARC). However, their associated toxicities negatively affect patients’ quality of life (QoL), prompting growing interest in exercise as a supportive intervention. This critical narrative review synthesised evidence on exercise interventions delivered before, during, or after NACRT/NART, with a focus on feasibility, safety, functional, psychosocial, and emerging oncological outcomes.

**Materials and methods:**

A systematic literature search was conducted across MEDLINE, CINAHL Ultimate, and SPORTDiscus (2010–2025). Prospective studies evaluating exercise-based interventions in patients with LARC receiving NACRT or NART were included. Studies were selected using the Participants, Exposure, and Outcome framework, and methodological quality was appraised using Critical Appraisal Skills Programme checklists. Data were synthesised narratively in accordance with PRISMA reporting standards.

**Results:**

Sixteen studies met the inclusion criteria. Interventions varied in type, intensity, supervision, and timing. Of these, nine studies (*n* = 226) were analysed quantitatively.

**Feasibility outcomes varied widely across studies:**

Eligibility (27.1–100%), recruitment (27.5–90.0%), attendance (74.0–96.0%), attrition (0–100%). No serious adverse events were reported. Consistent improvements were observed cardiorespiratory fitness, strength, fatigue, QoL. Limited evidence suggested a potential association between post-NACRT exercise and tumour regression; however, some of these findings were not powered for oncological endpoints.

**Interpretation:**

Exercise is a feasible and safe adjunct to treatment in LARC. Further research should explore oncological outcomes and improve representation across demographics.

## Introduction

Rectal cancer accounts for approximately one third of colorectal cancer diagnoses and is characterised by distinct anatomical, biological, and therapeutic considerations. Common presenting symptoms include rectal bleeding, altered bowel habits, abdominal pain, reduced appetite, and unexplained weight loss [1]. If left untreated, these may lead to severe complications, including mortality. In England, rectal cancer affects approximately 7486 individuals annually, with 43% of new diagnoses in those aged 75 or older [2]. Incidence is also increasing among adults under 50 years, reflecting a concerning epidemiological shift [3]. With cancer diagnoses projected to rise substantially by 2040 due to population ageing [2], evaluation of current and emerging management strategies is increasingly important.

Locally advanced rectal cancer (LARC) comprises approx-imately 54% of cases [4], and presents significant treatment challenges, particularly when tumours threaten resection margins on magnetic resonance imaging. Neoadjuvant radiotherapy (NART), often combined with chemotherapy (neoadjuvant chemoradiotherapy; NACRT), is standard care and aims to downstage tumours, facilitate clear surgical margins, and reduce local recurrence [4, 5]. Despite oncological benefit, neoadjuvant treatment is associated with substantial acute and longer-term toxicities, including fatigue, sarcopenia, reduced physical fitness, impaired cardiopulmonary function, diminished quality of life (QoL), and psychological distress [6, 7]. Postoperative recovery may be further complicated by poor wound healing and anastomotic leakage, with preoperative fitness emerging as a key determinant of outcomes [8]. Consequently, prehabilitation strategies aimed at optimising functional capacity before surgery are gaining prominence. Exercise-based interventions may mitigate treatment-related physiological decline, reduce fatigue, and enhance physical and cardiovascular function [9, 10].

A critical distinction between rectal and colon cancer lies in treatment sequencing. In the UK, colon cancer is typically managed with upfront surgical resection followed by adjuvant chemotherapy according to pathological stage, whereas rectal cancer commonly involves NART or chemoradiotherapy prior to surgery [11]. As a result, exercise interventions in colon cancer are usually delivered after completion of curative treatment with a rehabilitative or survivorship focus. In contrast, exercise delivered during neoadjuvant treatment for rectal cancer may interact with active oncological therapy and its associated physiological stressors, with important implications for feasibility, safety, and potential mechanisms of benefit.

From a radiobiological perspective, exercise may be particularly relevant during neoadjuvant therapy. Radiotherapy efficacy is strongly influenced by tumour hypoxia, a recognised contributor to radio-resistance in rectal cancer [12]). Preclinical and early-phase clinical studies suggest that aerobic exercise can transiently improve tumour perfusion and oxygenation, modulate the tumour microenvironment, and influence immune function, providing a plausible mechanistic rationale for synergy with NACRT [13, 14]. Clinically, approximately 13% of patients with LARC achieve a pathologically complete response, raising interest in non-operative management strategies in selected cases [15].

Despite growing interest, clinical implementation of exercise interventions during neoadjuvant rectal cancer treatment remains limited. Concerns regarding feasibility, safety, acceptability, and adherence persist, particularly for older patients and those with comorbidities. Additionally, heterogeneity in intervention design, frequency, duration, supervision, and outcome reporting has hindered evidence synthesis and translation into practice. During neoadjuvant therapy, patients often experience reduced autonomy due to intensive treatment schedules and symptom burden, making intervention characteristics such as exercise frequency and duration particularly relevant determinants of participation and retention.

Understanding how intervention characteristics relate to recruitment, attendance, and attrition is therefore essential to guide the development of future exercise protocols that balance potential physiological benefit with acceptability and commitment burden.

Examination of these relationships may help inform pragmatic, patient-centred trial design in an emerging and heterogeneous evidence base, rather than to infer causal effects.

This review therefore evaluates the feasibility, safety, and efficacy of exercise interventions delivered before, during, or after NACRT/NART in patients with LARC, focusing on functional, psychosocial, and emerging oncological outcomes. A secondary exploratory aim was to examine whether intervention frequency and duration appeared to be associated with recruitment, attendance, and attrition, in order to generate hypotheses and inform the design of future pragmatic, patient-centred exercise trials in an emerging and heterogeneous evidence base.

### Objective

This critical review synthesised the current evidence base to address five objectives:

1. To evaluate the feasibility of delivering exercise interventions before, during or after NACRT/NART in LARC
2. To assess the safety and tolerability of exercise interventions in this context
3. To determine the impact of exercise on functional and fitness-related outcomes
4. To explore emerging evidence relating to tumour response or oncological outcomes.
5. To examine behavioural and psychological factors associated with adherence and retention.

By systematically integrating findings across diverse study designs, this review aims to clarify the clinical role of exercise as a complementary intervention in LARC management and to inform future research priorities.

## Materials and methods

This review was designed as a critical narrative review with structured synthesis of feasibility and outcome data. Therefore, it synthesised relevant studies that incorporated exercise before or during NART or NACRT for rectal cancer, as well as during the treatment-free interval prior to surgery. As this review was based on previously published peer-reviewed studies, formal ethics approval was not required. Nonetheless, data collection and interpretation procedures adhered to ethical standards. In addition, this critical review was also conducted in accordance with the Preferred Reporting Items for Systematic Reviews and Meta-Analyses (PRISMA) guidelines [16]. This review was not prospectively registered on PROSPERO, given the feasibility-focused and narrative nature of the synthesis and the limited number of eligible studies, this is acknowledged as a limitation, which may increase the risk of reporting bias; however, we adhered to PRISMA guidelines to ensure transparency. A completed PRISMA 2020 checklist [17] is attached as a Supplementary Material.

### Inclusion and exclusion criteria

Eligibility was defined using the Participants, Exposure, and Outcome (PEO) framework [18]:

- **Participants**: Adults with LARC receiving or preparing for NART or NACRT. Randomised controlled trials (RCTs) and prospective cohort studies were included due to limited research.
- **Exposure**: Structured exercise interventions, defined as planned, repetitive bodily movement to improve or maintain health or fitness [19], delivered before, during, or after neoadjuvant treatment.
- **Outcomes**: Studies had to report at least one of:
  - Feasibility (eligibility, recruitment, attendance, retention)
  - Safety (adverse events, treatment interruptions)
  - Functional/physiological outcomes (cardiorespiratory fitness, strength, QoL) Studies focusing solely on surgical techniques, pharmacological therapies, or diet-only interventions were excluded.

### Search strategy

**Figure 1:**
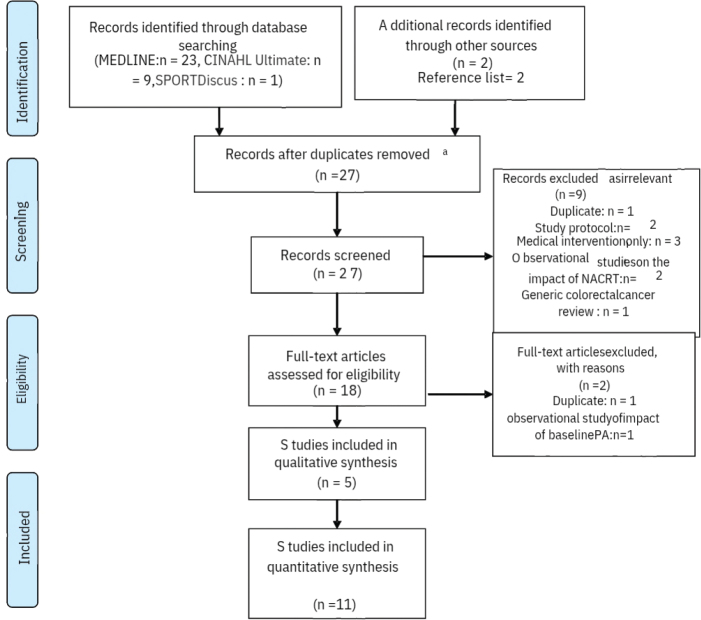
Adapted PRISMA flow diagram.

A structured search was conducted on the 15th of July 2025, updated search on the 21st of January 2026 by two reviewers (CA and SR) independently, using MEDLINE, CINAHL Ultimate, and SPORTDiscus via EBSCOhost for publications from 2010 to 2025. Reference lists of eligible studies were also hand-searched by one reviewer CA. The study selection was completed by the two reviewers, and the process is detailed in an adapted PRISMA flow diagram (Figure 1), with study characteristics presented in Table 1. Data extraction undertaken by one reviewer (CA). This approach was necessitated by resource constraints and is acknowledged as a limitation.

**Table 1:** Characteristics of included studies.

Authors (year) and study Design	Sample size (n)	Gender (M:F)	Age (mean)	Population	Setting and Supervision	Timing (Pre/During/Post NACRT)	Outcome measures	Intervention (Duration and Intensity)	Adverse events	Key results	CASP Grade
**Morielli et al. (2016) Phase I single-group feasibility study**	18	12:6	57.5	Adults (Stage II–III rectal cancer) receiving NACRT; ECOG ≤ 2; able to walk unaided	Cross Cancer Institute, Edmonton, Canada. Supervised + unsupervised aerobic sessions	During and Post	VO₂max, QoL, sleep, anxiety, adherence	6 weeks, 3 times per week supervised aerobic (moderate intensity, RPE 12–14), ~45 min/session; post-NACRT: ≥ 150 min/week unsupervised	None serious	Feasible with strong adherence; QoL improved	Moderate
**Morielli et al. (2016) Motivation Descriptive TPB sub-study**	18	12:6	57.5	Same cohort as above; included based on completion of NACRT and aerobic programme	Same cohort. Supervised HIIT + post-NACRT unsupervised with motivational themes	During and Post	TPB constructs, motivation, barriers, benefits	Same as above	None serious; mild fatigue, diarrhoea	Enjoyable, motivational support key to post-NACRT adherence	Moderate
**Morielli et al. (2018) Adherence pilot study**	18	12:6	57.5	Same cohort as above; subset selected for adherence analysis; inclusion based on programme completion	University of Alberta, Canada.	During and Post	Adherence metrics, TPB predictors	Same protocol as above	None serious; diarrhoea common	Adherence linked to gender, mental health, baseline PA	Moderate
**Morielli et al. (2021) Phase II RCT (EXERT Trial)**	36	24:12	57 ± 12	Stage II/III rectal cancer undergoing NACRT; able to tolerate HIIT; cleared for post-treatment aerobic follow-up	Edmonton, Canada. Supervised HIIT + unsupervised aerobic	During and Post	VO₂peak, pCR, toxicity, completion	6 weeks during NACRT: 3×/week supervised HIIT (treadmill), intervals at 85% VO₂peak, 25–30 min/session; post-NACRT: ≥ 150 min/week unsupervised aerobic	No serious issues	pCR ↑ in exercisers (56% vs 18%); feasible and safe	High
**Moug et al. (2019) -REx Trial, 2-arm RCT**	48	31:17	65.9	Rectal cancer patients aged > 60 scheduled for surgery post-NACRT; ambulatory and community dwelling	NHS Glasgow. Remote supervised walking prehabilitation	Before, During and Post	QoL, step count, sit-to-stand, depression	13–17 weeks walking programme; daily steps increased approx. 3000; intensity moderate (MET 10); unsupervised, session duration variable (self-paced)	None serious	Strong feasibility, adherence, QoL improvement	High
**Moug et al. (2020) Sub-analysis of REx Trial**	44	28:16	66.8	Subset of REx Trial patients with Pre-/post-abdominal CT scans; inclusion based on full body imaging	Subgroup with CT-based TPI analysis	Before and During	Muscle mass (TPI), sarcopenia	Same graduated walking protocol as above	None serious	Muscle mass ↑ in intervention group; TPI protective effect	High
**Mast et al. (2025) 3-arm RCT feasibility trial**	3	Unreported	Unreported	Adults with locally advanced rectal cancer undergoing NACRT; eligible for structured hospital exercise post-treatment	Radboud UMC Netherlands. Hospital-based aerobic (ExPR), AE+RE vs control	During and Post	VO₂max, tumour response, toxicity	5 weeks. ExPR: 5×/week supervised cycling at CPET-based intensity, 30–40 min/session; AE+RE: 2×/week mixed exercise + 1×/week home, approx. 60 min/session	Minor fatigue, back pain	VO₂max ↑; tumour response ≥ 70%; reduced toxicity	Low
**Piraux et al. (2022) 3-arm RCT feasibility study**	18	13:5	62.0	Rectal cancer patients completing radiotherapy; medically stable and cleared for exercise by oncology team	Brussels. HIIT, resistance, usual care arms	During	QoL, fatigue, cognition, sleep	5 weeks, 3×/week supervised sessions. HIIT: ≥ 85% HRmax intervals, approx. 30–35 min/session; RES: moderate-load circuits, approx. 40 min/session	Diarrhoea, fatigue; no withdrawals	Attendance 88–92%; comparable improvements; RES ↑ social wellbeing	Moderate–High
**Felipe et al. (2019) Single-armpilot**	12	3:9	61 ± 7	Stage II–III rectal cancer patients mid-NACRT; cognitively able to participate in education sessions and park-based activity	Madrid. Community-delivered exercise education	During	VO₂ peak, QoL, HADS, fitness tests	5 weeks, 6 sessions total (1–2×/week), approx. 60 min/session; moderate-to-vigorous group-based aerobic/resistance/flexibility	None serious; 1 dropout	VO₂peak ↑, MVPA ↑, depression ↓	Low
**Brunet et al. (2021) Feasibility trial (not delivered)**	10 referred, 0 enrolled	n/a	n/a	Intended: Adults with rectal cancer scheduled for NACRT and surgery at Ottawa Hospital; eligible for supervised trial	Ottawa Hospital, Canada. Planned supervised	During	Recruitment and feasibility barriers	Planned: 12 weeks, 3×/week supervised aerobic + resistance at 60–75% HRR, ~45–60 min/session (not delivered)	Not delivered	Study infeasible; eHealth and hybrid models recommended	Low
**West et al. (2015) Non-Randomised Controlled interventional pilot**	39	27:12	64 (Ex); 72 (Control)	Patients with locally advanced rectal cancer completing NACRT; eligible for post-NACRT cycling based on CPET clearance	UK. CPET-Supervised structured cycling sessions	Post	VO₂LT, VO₂peak, step count	6 weeks, 3×/week CPET-responsive cycling (threshold and peak VO₂), approx. 40 min/session	None serious	VO₂LT improved in exercisers; reversed NACRT fitness decline	High
**Loughney et al. (2017) Non-Randomised Controlled pilot**	39	27:12	64 (Ex); 72 (Control)	Same cohort as above; subset with valid accelerometry data; inclusion based on consent and fitness screening	UK. Same cohort as West 2015	Post	PAL, sleep metrics, EE, MET	Same as above	None serious	Sleep efficiency ↑; MET recovery in exercise group	High
**West et al. (2019) Mechanistic exploratory study**	35	26:9	64.8 (Ex); 70.2 (Control)	Patients with MRI-defined threatened margins post-NACRT; included if eligible for prehabilitation and MRI follow-up	UK. As above. MRI-defined margin-threatened cases	Post	TRG, VO₂LT, CPET, tumour regression	Same as above	None serious	Tumour regression ↑ in exercisers (OR 8.5); VO₂ restored	High
**Loughney et al. (2021) (EMPOWER Trial) Multicentre RCT**	33	26:7	64 ± 10.4	Adults with rectal cancer post-ACRT, margin-threatened disease, CPET cleared	UK. 5 NHS sites; supervised post-NACRT programme	Post	VO₂LT, HRQoL, PA, interviews	9 weeks, 3×/week supervised CPET-responsive programme; progressive intervals; 40 min/session	1 pre-syncope; no serious	VO₂LT ↑; sychologic al resilience ↑ Post-NACRT VO2 at AT ↓significantly in all participants	High
**Singh et al. (2018) Feasibility study**	10	7:3	54.6 ± 14.1	Adults with localised rectal cancer undergoing NACRT; medically stable and willing to attend supervised sessions	Perth, Australia. Resistance + aerobic programme during CRT	During	Muscle strength, QoL, DXA lean mass, fatigue	10 weeks; 2×/week supervised resistance + aerobic at 60–80% HRmax, approx. 60 min/session; plus 2×/week unsupervised aerobic (30–45 min)	None serious	Muscle strength ↑, ASM loss ↓; QoL preserved	Moderat
**Heldens et al. (2016) Prospective single-arm pilot**	13 (9 completed)	11:2	59.1 ± 19.7	Adults with locally advanced resectable rectal cancer receiving NACRT; no contraindications to moderate exercise	Maastricht, Netherlands. Outpatient supervised programme	During	Muscle strength, 6MWT, fatigue, SF-36	9–17weeks, 2×/week supervised moderate-intensity aerobic + resistance (Borg 13–14), 45–60 min/session	None serious	Leg ↑39.2%, arm ↑34.9%, fatigue stable slight ↑ 6MWT; feasible with high satisfaction	Moderat

QoL: Quality of Life; SF-36: Short-Form 36 Health Survey; EQ-5D-5L: EuroQol 5 Dimensions, 5 Levels; HADS: Hospital Anxiety and Depression Scale; FACIT-F: Functional Assessment of Chronic Illness Therapy-Fatigue; FACT-G: Functional Assessment of Cancer Therapy-General; CES-D: Center for Epidemiologic Studies–Depression Scale; ESS: Epworth Sleepiness Scale; ISI: Insomnia Severity Index; PSQI: Pittsburgh Sleep Quality Index; MFI: Multidimensional Fatigue Index; TPB: Theory of Planned Behaviour; HIIT: high-intensity interval training; 6MWT: 6-minute walk test; DXA: Dual-energy X-ray Absorptiometry; CRT: Neoadjuvant chemoradiation treatment

The search used Boolean operators across three thematic blocks:

- rect* cancer OR rect* neoplasm OR LARC OR LARC AND
- resistance training OR aerobic exercise OR aerobic training OR physical activity OR exercise OR physical exercise

AND

- NACRT OR NART OR short course NART OR long course NACRT

### Quality assessment

Evaluating the methodological quality of included studies is essential for robust literature reviews [20]. Methodological quality was assessed using the Critical Appraisal Skills Programme (CASP) checklists appropriate to study design. Studies were not excluded based on quality due to the limited number of studies and the focus on feasibility; rather, appraisal findings regarding, relevance, reliability, validity, and applicability informed interpretation of the evidence. Domains evaluated included sample size calculations, randomisation methods, blinding, baseline group similarity, dropout rates, adherence, outcome measure validity, and pre-specified outcomes. CASP tools are recognised for their reliability in assessing bias in cohort studies and RCTs.

Studies were graded as low, moderate, or high quality. Sample representativeness, disease progression stage, and participant homogeneity (age, body mass index, ethnicity) were also considered. Quantitative feasibility analysis was performed using IBM SPSS version 29 (SPSS Inc., Chicago, IL, USA).

### Data extraction and analysis

Data from eligible studies were extracted using a standardised IBM SPSS template, capturing:

- Study design, country, sample size, and demographics
- Exercise intervention details (mode, intensity, frequency, supervision)
- Eligibility rate, attendance/adherence rate, and dropout rates
- Physical activity/fitness outcomes (such as, VO₂max, heart rate reserve, target maximal heart rate, 6-minute walk test, muscle mass)
- Safety incidents and adverse events
- Psychosocial outcomes (QoL, fatigue, motivation)

### Data synthesis and exploratory analyses

Data were synthesised narratively, grouping findings into themes: feasibility and adherence, safety, functional and fitness outcomes, psychosocial effects, predictors of adherence, and oncologic/tumour-related outcomes. This was due to substantial clinical and methodological heterogeneity across studies, including variation in intervention timing, modality, intensity, supervision, and outcome measures [21, 22]. To complement narrative synthesis and support future protocol development in exercise oncology, limited exploratory quantitative analyses were undertaken using study-level aggregated data explicitly reported in the included studies. Specifically, Pearson’s correlation coefficients were calculated to examine linear associations between prescribed exercise frequency (sessions per week), intervention duration (number of weeks), and feasibility outcomes including recruitment, attendance, and attrition. These analyses were conducted for hypothesis-generating purposes only and were not intended to establish causal relationships. This approach is consistent with prior methodological work in feasibility research and complex intervention development, where exploratory analyses of aggregated trial-level data have been used to inform intervention design in emerging [23, 24]. Standardised 95% confidence intervals were reported, with an alpha value of 0.05 for statistical significance.

## Results

### Database search

The database search yielded 25 abstracts after removing duplicates (*n* = 8) MEDLINE (23 studies), CINAHL Ultimate (9 studies), and SPORTDiscus (1 study). Reference screening added two studies [25, 26]. After title and abstract screening, nine studies were excluded for reasons including duplication (*n* = 1), protocol/methodological papers (*n* = 2), general colorectal cancer focus without rectal cancer subgroup (*n* = 1), observational studies without exercise interventions (*n* = 2), or non-exercise medical interventions (*n* = 3). Eighteen studies underwent full-text review, with two further excluded for duplicated data or observational design. Sixteen studies, involving 226 participants with LARC receiving or recovering from NACRT, were included in the final synthesis. Study selection is summarised in the PRISMA flow diagram (Figure 1), and study characteristics are presented in Table 1.

Eleven studies used interventional designs, including four RCTs or pilot RCTs. Studies spanned seven countries (England, Scotland, Australia, Netherlands, Canada, Belgium, Spain) and included inpatient and home-based interventions across NACRT, post-NACRT, or pre/postoperative phases. Nine studies implemented exercise thrice weekly, four twice weekly, with durations ranging from 5 weeks [27–29] to 17 weeks [30, 31]. Most delivered exercise during chemoradiotherapy, with one study [26] specifying post-radiotherapy sessions. Study characteristics are summarised in Table 1. Although three of the included studies were rated as low quality according to CASP [27, 29, 32] their inclusion did not materially alter findings significantly.

### Patients’ characteristics

Patient characteristics are detailed in Table 2. Two studies [29, 32] lacked demographic data due to poor recruitment. Among the remaining studies, mean or median age and sex distribution were consistently reported, with a male-to-female ratio of approximately 2.4:1 across the studies. Reporting of Body Mass Index (BMI) and ethnicity was inconsistent, with one study omitting BMI [7] and another not reporting female representation [26].

**Table 2:** Cumulative sex characteristics of included studies.

Patient characteristics	Minimum	Maximum	Mean	Std. deviation	Skewness	
					Statistic	Std. error
Male	0	31	15.80	11.361	0.135	0.687
Female	0	17	7.33	5.568	0.436	0.717

### Feasibility outcomes

Feasibility outcomes were synthesised descriptively at the individual study level and are presented in Table 3. Recruitment rates varied widely across studies, reflecting differences in eligibility criteria, timing relative to neoadjuvant therapy, and recruitment settings. Studies embedding exercise delivery within routine clinical care or providing supervised sessions generally reported higher recruitment and retention than those relying solely on self-directed or home-based programmes.

**Table 3:** Feasibility metrics across included studies.

Study	Eligibility rate (%)	Recruitment rate (%)	Attrition rate (%)	Adherence/attendance rate (%)	Decline rate (%)
Morielli, Usmani [35]	71.11	56.25	22.22	74.00	43.75
Morielli, Boulé [25]	63.90	27.48	63.89	82.00	73.00
Moug, Mutrie [30]	100.00	61.54	16.67	75.00	38.46
Mast, Gootjes [29]	Unreported	14.29	0	Unreported	85.71
Piraux, Reychler [28]	86.96	90.00	0	92.00	10.00
Felipe, Alejo [27]	82.14	52.17	16.67	89.00	Unreported
Brunet, Price [32]	85.70	50.00	100.00	Unreported	Unreported
West, Loughney [7]	Unreported	Unreported	10.26	96.00	Unreported
Loughney, West [33]	27.08	48.72	34.21	91.00	51.28
Heldens, Bongers [31]	Unreported	65.00	30.77	95.70	33.33
Singh, Galvão [26]	Unreported	66.67	30.00	77.00	25.00

Attendance and adherence were reported using hetero-geneous metrics, including proportion of prescribed sessions attended, weekly exercise minutes achieved, or categorical adherence thresholds. Direct comparison across studies was therefore not possible.

### Eligibility and recruitment

Eligibility rates varied widely across studies, ranging from 27.1 [33] to 100% [30], reflecting differences in inclusion criteria, clinical pathways, and recruitment settings. Of the 417 eligible individuals, 226 were enrolled, yielding an overall recruitment rate of 54.2%.

Recruitment ranged from 27.5 to 90% across studies. Recruitment challenges were common, with two studies terminating early due to insufficient enrolment [29, 32]. Studies integrating exercise delivery within routine oncology care or offering supervised sessions tended to achieve higher recruitment.

### Attendance and adherence

Adherence was reported using heterogeneous metrics, including proportion of prescribed sessions attended, weekly exercise minutes achieved, or categorical adherence thresholds. Despite this variability, attendance was generally high, ranging between 74.0 and 96.0% across studies. Supervised hospital-based interventions demonstrated the highest adherence (91% in Loughney, West [33]; 98% in West, Astin [9]). Home-based programmes showed more variable adherence, though the REX trial [30] achieved 75% adherence with weekly telephone support. Predictors of adherence included younger age, female sex, marital status, higher baseline motivation, and fewer gastrointestinal side effects [34].

### Exercise-based intervention attrition rates

Attrition rates again varied widely across studies (0–100%), averaging 32.5% (SD = 29.3%), with primary reasons including treatment-related toxicity, disease progression, and logistical barriers such as travel distance [9, 35]. No study reported attrition attributable to exercise-related harm.

### Exploratory associations between intervention characteristics and feasibility metrics

To inform future trial design, exploratory analyses examined associations between intervention characteristics and feasibility outcomes using study-level aggregated data explicitly reported in individual publications; these are summarised in Table 4. A moderate positive association was observed between intervention duration and attrition (*r* = 0.433), suggesting that longer programmes may be associated with increased participant withdrawal during neoadjuvant treatment. A weak negative association was observed between prescribed exercise frequency and recruitment (*r* = −0.263), indicating that higher-frequency prescriptions may pose a barrier to initial participation. No clear association was observed between intervention characteristics and session attendance. These findings were not statistically significant and are reported for hypothesis-generation only, to inform future research rather than to support causal interpretation.

**Table 4:** Impact of exercise duration and frequency on recruitment rate, attendance, and attrition.

Feasibility Measures	Correlation study	Intervention duration	Intervention frequency
Recruitment rate	Pearson correlation	-0.029	-0.263
	Sig. (2-tailed)	0.941	0.529
Attrition rate	Pearson correlation	0.433	0.071
	Sig. (2-tailed)	0.212	0.855
Attendance	Pearson correlation	-0.382	-0.067

### Safety and tolerability

No serious adverse events attributable to exercise participation were reported. Safety monitoring methods varied across studies. Supervised interventions commonly employed pre-session symptom screening, real-time monitoring by qualified professionals, and predefined criteria for exercise modification or cessation. Home-based interventions primarily relied on participant self-report with clinical follow-up contact and escalation pathways when required. No studies reported exercise-related interruptions to neoadjuvant therapy or delays to surgery. Minor symptoms (fatigue, skin irritation, gastrointestinal discomfort) occurred but did not cause withdrawals or treatment interruptions. Real-time monitoring (such as, Borg scale, CPET titration, physiotherapist supervision) ensured safety.

### Intervention modalities and delivery

Exercise modalities included:

- **Aerobic Training**: Walking, cycling, or treadmill-based, ranging from moderate to high-intensity interval training (HIIT) [25, 28].
- **Resistance Training**: Used in five studies, standalone or combined with aerobic exercise [26, 28].
- **Multicomponent Education-Led Programmes**: Community-based exercise education showed promising adherence and QoL improvements [27]. Supervised hospital-based [7, 33], remote, or hybrid delivery models ensured fidelity and flexibility.

### Impact on functional capacity and fitness

Cardiorespiratory fitness (VO₂AT) declined during NACRT but improved post-exercise interventions, with gains of 2.1–2.9 ml/kg/min in trials [7, 33]. The 6-minute walk test and step counts showed positive trends (+69m in [30]). Resistance training improved leg (+39%) and arm strength (+35%) [31] and muscle mass (+16 mm²/m² [36]). However, Heldens, Bongers [31] and West, Loughney [7] found that some preoperative improvements were not maintained postoperatively, with three participants in the Heldens, Bongers [31] trial experiencing declines to below baseline levels.

### Psychosocial and behavioural outcomes

Seven studies reported psychosocial benefits, including improved QoL, reduced fatigue, and lower depression scores [33, 35]. Participants enjoyed exercise more than expected (*p* = 0.003), citing enhanced self-esteem and physical function. Predictors of positive psychosocial response included younger age, female sex, and fewer treatment-related side effects. Motivation commonly declined after NACRT, highlighting the need for tailored behavioural support [34].

### Oncological and tumour-related outcomes

Three studies reported oncologic outcomes. West, Astin [9] found improved tumour regression grading (*p* = 0.02) post-aerobic intervention, although this analysis was exploratory and the original study was not powered for oncological endpoints. R Morielli, Usmani [37] reported a higher pathological complete response rate (39% vs. 12%, *p* = 0.020) in the exercise group. Mast et al. [29] did not report oncological outcomes for rectal cancer due to recruitment limitations. Overall, evidence for oncological benefit remains preliminary but suggests potential biological relevance warranting further investigation.

## Discussion and conclusion

This critical review synthesised evidence from 16 studies evaluating the feasibility, safety, and efficacy of exercise interventions delivered during or around NART and NACRT for patients with LARC. Collectively, the findings indicate that exercise interventions are generally feasible and safe in this population, with consistent benefits for physical function and QoL. However, substantial heterogeneity in study design, intervention characteristics, and outcome reporting continues to limit definitive conclusions and highlights important priorities for future research.

Most studies included in this review were of high methodological quality per CASP assessments but were limited by small sample sizes, typical of feasibility trials, constraining statistical power and generalisability [38]. The largest RCT [30], with 49 participants, was underpowered for definitive conclusions on efficacy.

In addition, the lack of reporting on ineligible participants in most studies impedes understanding of barriers to inclusion and limits refinement of intervention accessibility. Future studies should ensure transparent reporting of inclusion barriers to enhance inclusive intervention design.

Across studies, feasibility outcomes demonstrated both promise and challenge. Recruitment rates ranged from approximately 56–65% in single-centre studies [31, 35] and higher success in multicentre trials [33]. In contrast, poor recruitment reported by Brunet, Price [32] despite a year-long recruitment period, stemmed from logistical issues such as transport and lack of clinical integration. Overall, these findings suggest that feasibility is strongly influenced by how closely exercise delivery aligns with routine clinical care. However, addressing logistical barriers, such as transportation and referral pathways, is essential to optimise real-world implementation [7, 26, 31, 33, 39].

Adherence and attendance were generally favourable, particularly in supervised interventions, and were often supported by positive patient experiences. Participants frequently reported greater enjoyment than anticipated and perceived benefits in self-esteem, physical function, and QoL [34]. These findings align with broader colorectal cancer literature, including a systematic review by Singh, Hayes [40], who reported an average adherence rate of 86% in colorectal cancer exercise trials. Nevertheless, treatment-related fatigue, gastrointestinal symptoms, and motivational decline following NACRT remained important barriers, particularly in unsupervised programmes.

The Colon Health and Life-Long Exercise Change (CHALLENGE) trial Courneya Kerry, Vardy Janette [41] provides valuable contextual insight into how behavioural support may mitigate such barriers. Although conducted in colon cancer survivors following adjuvant chemotherapy, CHALLENGE demonstrated that a theory-driven, behaviourally supported exercise programme could sustain adherence over several years, despite ongoing fatigue and deconditioning. These findings reinforce that exercise prescriptions require more than generic advice; structured behavioural strategies, supervision, and follow-up are central to long-term engagement. While CHALLENGE is not directly transferable to rectal cancer populations undergoing neoadjuvant therapy, it offers an important benchmark for intervention quality and imple-mentation.

Rejection rates across the studies were substantial, ranging from 10 to 85.7% [28, 29], reflecting treatment burden, emotional distress, and competing clinical demands. Exploratory analyses in this review suggested a moderate association between longer intervention duration and higher attrition, alongside a weak negative association between prescribed exercise frequency and recruitment. Although these analyses were hypothesis-generating and not statistically inferential, they provide a rationale for considering intervention burden as a modifiable design feature. During neoadjuvant treatment, when many aspects of care feel externally controlled, overly intensive or inflexible exercise prescriptions may exacerbate perceived burden and deter participation.

Mode of delivery also appeared influential. Supervised hospital-based programmes were associated with higher attendance and greater improvements in cardiorespiratory fitness compared with unsupervised approaches [7, 42]. However, home-based and remote interventions demonstrated acceptable feasibility in several studies [27, 36]. suggesting that hybrid delivery models may offer a pragmatic balance between effectiveness and accessibility. Such models may be particularly relevant for patients facing transport challenges or treatment-related fatigue.

Safety outcomes across the included studies were consistently reassuring. Except for Felipe, Alejo [27], which did not report safety outcomes, no serious exercise-related adverse events were identified. This aligns with wider oncology exercise literature reporting low adverse event rates during neoadjuvant therapy [43, 44]. Minor events such as transient fatigue, musculoskeletal discomfort, or presyncope were infrequent and did not result in withdrawal [31, 33]. However, approaches to safety monitoring varied considerably, ranging from passive self-report to structured clinician-led assessment using instruments such as patient or reported version of the Common Terminology Criteria for Adverse Events (PRO-CTCAE) [29]. This heterogeneity limits comparability and highlights the need for standardised safety reporting in future trials.

Exercise interventions were associated with consistent improvements in physical function and QoL. Gains in cardiorespiratory fitness, including increases in ventilatory threshold of 2.1–2.9 ml/kg/min, were reported in several studies [7], alongside attenuation of treatment-related functional decline and ameliorate physical and functional decline [7, 25, 26, 35]. However, meta-analytic evidence from Zhang [45] found no significant improvements in functional capacity as measured by the 6-minute walk test (6MWT), underscoring the potential influence of intervention timing. Zhang [45] found no significant improvement in 6-minute walk distance, suggesting that outcome sensitivity and intervention timing may influence observed effects. Prehabilitation prior to NACRT remains underexplored, with only one study addressing this phase [30] despite evidence that physical activity declines during neoadjuvant treatment [6, 46] and that higher baseline activity levels are associated with improved tumour downstaging [8, 47].

Emerging evidence also suggests a potential oncological role for exercise. Two studies reported significantly greater tumour regression among patients undertaking structured exercise following NACRT, representing the first human data in rectal cancer to suggest that exercise may augment tumour response [9, 25]. While these findings are preliminary and include post hoc analyses not powered for oncological endpoints, they are biologically plausible. Preclinical and early clinical studies indicate that exercise may improve tumour perfusion, modulate immune activity, and reduce hypoxia, mechanisms directly relevant to radiotherapy efficacy [13, 14, 48, 49]. Further mechanistic investigation is warranted.

The relevance of survivorship evidence from colon cancer must be interpreted cautiously. The CHALLENGE trial [41] demonstrated a statistically significant improvement in 5-year disease-free survival, corresponding to a 28% reduction in recurrence risk, establishing exercise as a disease-modifying intervention in colon cancer. However, rectal cancer management differs fundamentally, particularly in the UK, where NART or chemoradiotherapy is standard [11]. These differences have implications for feasibility, safety, and the mechanisms through which exercise may exert benefit, reinforcing the need for rectal cancer-specific trials rather than extrapolation from survivorship cohorts.

Intervention modality also warrants consideration. The HIIT has emerged as a time-efficient and tolerable option during NACRT, offering comparable or superior cardiorespiratory benefits relative to moderate-intensity continuous training [25, 28]. Resistance training remains underrepresented despite evidence of benefits for muscle mass and strength, outcomes particularly relevant for sarcopenia and cachexia prevention [26, 36].

Finally, the evidence base is limited by under-representation of women, ethnic minority groups, older adults, and patients with multimorbidity. Only 4% of participants in a large UK feasibility study were non-Caucasian [30], limiting generalisability. No studies evaluated exercise during NART alone, despite its relevance for frail patients [50]. Future research should therefore prioritise inclusive, pragmatic trial designs informed by successful large-scale exercise oncology studies in related populations. The CHALLENGE trial [41] demonstrated the feasibility of delivering a structured, behaviourally supported exercise intervention across six countries in 889 colon cancer survivors, achieving sustained adherence, and improvements in cardiorespiratory fitness. Addressing these gaps will be essential to ensure equitable and scalable integration of exercise into rectal cancer care.

In summary, exercise interventions appear feasible and safe for many patients with LARC undergoing neoadjuvant treatment, with meaningful benefits for physical function and QoL. Future research should prioritise adequately powered, inclusive trials with standardised reporting, mechanistic endpoints, and patient-centred intervention design to clarify the role of exercise as a supportive and potentially treatment-modifying therapy in rectal cancer.

### Future research directions

- Evaluate exercise during NART for feasibility and efficacy in frail populations
- Investigate optimal intervention timing (e.g. pre-NACRT or post-radiotherapy)
- Develop inclusive recruitment strategies for more diverse populations
- Explore barriers (such as, fatigue, motivation) through qualitative research
- Replicate findings on exercise enhancing tumour response
- Ensure transparent reporting of eligibility and participation metrics

## Limitations

This review has several methodological limitations that should be considered when interpreting the findings. Firstly, study screening and study selection were conducted independently by two reviewers, which strengthened methodological rigour and reduced the risk of selection bias. However, data extraction and synthesis were undertaken by a single reviewer due to resource constraints. While this approach is commonly adopted in feasibility-focused narrative reviews, it may introduce a degree of interpretive bias, and future reviews would benefit from duplicate data extraction.

Secondly, the review protocol was not prospectively registered on PROSPERO. While protocol registration is recommended to enhance transparency and reduce the risk of reporting bias, this review was conceived as a feasibility-focused critical narrative synthesis within an emerging and heterogeneous evidence base. Nonetheless, the absence of prospective registration should be acknowledged as a limitation. To mitigate this risk, the review adhered closely to PRISMA reporting standards, and a completed PRISMA 2020 checklist is provided as supplementary material.

Thirdly, substantial clinical and methodological heterogeneity across included studies precluded meta-analysis. Heterogeneity was evident in intervention timing (before, during, or after neoadjuvant therapy), exercise modality, intensity, frequency, duration, level of supervision, and outcome measurement. As a result, findings were synthesised narratively, and comparisons across studies should be interpreted with caution.

Fourthly, exploratory analyses examining associations bet-ween intervention characteristics (exercise frequency and intervention duration) and feasibility outcomes were based on aggregated study-level data rather than individual participant data. These analyses were undertaken to generate hypotheses and inform future trial design, not to infer causality. The small number of studies, inconsistent reporting of feasibility metrics, and a lack of adjustment for potential confounders limit the interpretability and generalisability of these findings.

Fifthly, reporting of adverse events and safety monitoring varied across studies. Although no serious exercise-related adverse events were reported, differences in monitoring approaches, ranging from active supervision with structured symptom screening to reliance on participant self-report, may have resulted in under-reporting of minor adverse events, particularly in home-based interventions.

Finally, the included studies were characterised by relatively small sample sizes and limited demographic diversity, with under-representation of older adults, individuals with significant comorbidity, women, and ethnically or socioeconomically diverse populations. These factors limit the generalisability of findings and highlight the need for inclusive, adequately powered rectal cancer-specific exercise trials embedded within routine clinical care.

## Conclusion

The findings of this study consistently support exercise, particularly aerobic and resistance modalities, is feasible, safe, and beneficial for LARC patients undergoing NACRT, improving function and potentially tumour response. Small samples, demographic biases, and lack of NART-specific studies limit generalisability. Rectal cancer-specific trials are required to define optimal exercise prescription during neoadjuvant therapy and to further explore potential interactions with radiotherapy response. Inclusive recruitment, better reporting, and clinical integration are essential to optimise exercise in rectal cancer care.

## Implications for rehabilitation

Exercise can safely enhance health and function in LARC management but remains underutilised. Future interventions should target diverse populations, including women, ethnic minorities, and older adults, to ensure equitable benefits.

## Supplementary Material



## Data Availability

Data were derived from published studies listed in the references.

## References

[1] Briggs NL, Ton M, Malen RC, Reedy AM, Cohen SA, Phipps AI, et al. Colorectal cancer pre-diagnostic symptoms are associated with anatomic cancer site. BMC Gastroenterol. 2024;24(1):65. 10.1186/s12876-024-03152-8 38317073 PMC10845784

[2] 2. Office for National Statistics. National population projections: 2022-based. Statistical bulletin, (2025). https://www.ons.gov.uk/peoplepopulationandcommunity/populationandmigration/populationprojections/bulletins/nationalpopulationprojections/2022based

[3] Quinn TJ, Kabolizadeh P. Rectal cancer in young patients: incidence and outcome disparities. J Gastrointest Oncol. 2020;11(5):880–93. 10.21037/jgo-20-197 33209484 PMC7657826

[4] Kaul S, Rao C, Mane R, Tan KL. Is the management of rectal cancer using a watch and wait approach feasible, safe and effective in a publicly funded general hospital? Clin Oncol. 2022;34(1):e25–34. 10.1016/j.clon.2021.08.004 34454807

[5] Glynne-Jones R, Wyrwicz L, Tiret E, Brown G, Rödel C, Cervantes A, et al. Rectal cancer: ESMO Clinical Practice Guidelines for diagnosis, treatment and follow-up. Ann Oncol. 2017;28(Suppl_4):iv22–40. 10.1093/annonc/mdx224 28881920

[6] West MA, Loughney L, Lythgoe D, Barben CP, Adams VL, Bimson WE, et al. The effect of neoadjuvant chemoradiotherapy on whole-body physical fitness and skeletal muscle mitochondrial oxidative phosphorylation in vivo in locally advanced rectal cancer patients – an observational pilot study. PLoS One. 2014;9(12):e111526. 10.1371/journal.pone.0111526 25478898 PMC4257525

[7] West MA, Loughney L, Lythgoe D, Barben CP, Sripadam R, Kemp GJ, et al. Effect of prehabilitation on objectively measured physical fitness after neoadjuvant treatment in preoperative rectal cancer patients: a blinded interventional pilot study. Br J Anaesth. 2015;114(2):244–51. 10.1093/bja/aeu318 25274049

[8] Mast IH, de Wilt JHW, Duman B, Smit KC, Gootjes EC, Vissers PAJ, et al. Physical activity at diagnosis is associated with tumor downstaging after neoadjuvant chemoradiotherapy in patients with rectal cancer. Radiother Oncol. 2024;200:110523. 10.1016/j.radonc.2024.110523 39265927

[9] West MA, Astin R, Moyses HE, Cave J, White D, Levett DZH, et al. Exercise prehabilitation may lead to augmented tumor regression following neoadjuvant chemoradiotherapy in locally advanced rectal cancer. Acta Oncol. 2019;58(5):588–95. 10.1080/0284186X.2019.1566775 30724668

[10] Burke SM, Brunet J, Sabiston CM, Jack S, Grocott MPW, West MA. Patients’ perceptions of quality of life during active treatment for locally advanced rectal cancer: the importance of preoperative exercise. Support Care Cancer. 2013;21(12):3345–53. 10.1007/s00520-013-1908-2 23912669

[11] National Institute for Health and Care Excellence. Colorectal cancer. (NICE Guideline NG151) 2020. https://www.nice.org.uk/guidance/ng151 32813481

[12] Horsman MR, Overgaard J. The impact of hypoxia and its modification of the outcome of radiotherapy. J Radiat Res. 2016;57(Suppl 1):i90–8. 10.1093/jrr/rrw007 26983987 PMC4990104

[13] Dethlefsen C, Hansen LS, Lillelund C, Andersen C, Gehl J, Christensen JF, et al. Exercise-induced catecholamines activate the hippo tumor suppressor pathway to reduce risks of breast cancer development. Cancer Res. 2017;77(18):4894–904. 10.1158/0008-5472.CAN-16-3125 28887324

[14] Pedersen L, Idorn M, Olofsson GH, Lauenborg B, Nookaew I, Hansen RH, et al. Voluntary running suppresses tumor growth through epinephrine- and IL-6-dependent NK cell mobilization and redistribution. Cell Metab. 2016;23(3):554–62. 10.1016/j.cmet.2016.01.011 26895752

[15] Lorimer PD, Motz BM, Kirks RC, Boselli DM, Walsh KK, Prabhu RS, et al. Pathologic complete response rates after neoadjuvant treatment in rectal cancer: an analysis of the national cancer database. Ann Surg Oncol. 2017;24(8):2095–103. 10.1245/s10434-017-5873-8 28534080

[16] Moher D, Liberati A, Tetzlaff J, Altman DG. Preferred reporting items for systematic reviews and meta-analyses: the PRISMA statement. PLoS Med. 2009;6(7):e1000097. 10.1371/journal.pmed.1000097 19621072 PMC2707599

[17] Page MJ, McKenzie JE, Bossuyt PM, Boutron I, Hoffmann TC, Mulrow CD, et al. The PRISMA 2020 statement: an updated guideline for reporting systematic reviews. BMJ. 2021;372:n71. 10.1136/bmj.n71 33782057 PMC8005924

[18] Khan, K., R. Kunz, J. Kleijnen, and G. Antes, Systematic reviews to support evidence-based medicine, 2nd edition. London 2011, CRC Press.

[19] Garber CE, Blissmer B, Deschenes MR, Franklin BA, Lamonte MJ, Lee IM, et al. Quantity and quality of exercise for developing and maintaining cardiorespiratory, musculoskeletal, and neuromotor fitness in apparently healthy adults: guidance for prescribing exercise. Med Sci Sports Exerc. 2011;43(7):1334–59. 10.1249/MSS.0b013e318213fefb 21694556

[20] Aveyard H, Payne S, Preston N. A post-graduate’s guide to doing a literature review in health and social care. Maidenhead: Open University Press; 2016.

[21] Petticrew M, Roberts H. Systematic reviews in the social sciences: a practical guide. Oxford: Blackwell Pub; 2006.

[22] Higgins JPT, Thomas J, Chandler J, Cumpston M, Li T, Page MJ, et al, editor(s). Cochrane Handbook for Systematic Reviews of Interventions. 2nd Edition. Chichester (UK) 2019: John Wiley & Sons.

[23] Craig P, Dieppe P, Macintyre S, Michie S, Nazareth I, Petticrew M. Developing and evaluating complex interventions: the new Medical Research Council guidance. BMJ. 2008;337:a1655. 10.1136/bmj.a1655 18824488 PMC2769032

[24] Eldridge SM, Lancaster GA, Campbell MJ, Thabane L, Hopewell S, Coleman CL, et al. Defining feasibility and pilot studies in preparation for randomised controlled trials: development of a conceptual framework. PLoS One. 2016;11(3):e0150205. 10.1371/journal.pone.0150205 26978655 PMC4792418

[25] Morielli AR, Boulé NG, Usmani N, Tankel K, Joseph K, Severin D, et al. Effects of exercise during and after neoadjuvant chemoradiation on symptom burden and quality of life in rectal cancer patients: a phase II randomized controlled trial. J Cancer Surviv. 2023;17(4):1171–83. 10.1007/s11764-021-01149-w 34841461

[26] Singh F, Galvão DA, Newton RU, Spry NA, Baker MK, Taaffe DR. Feasibility and preliminary efficacy of a 10-week resistance and aerobic exercise intervention during neoadjuvant chemoradiation treatment in rectal cancer patients. Integr Cancer Ther. 2018;17(3):952–9. 10.1177/1534735418781736 29888608 PMC6142076

[27] Felipe JL, Alejo LB, Pagola-Aldazabal I, Lucia A, Fiuza-Luces C, Huerga D, et al. Exercise prehabilitation program for patients under neoadjuvant treatment for rectal cancer: a pilot study. J Cancer Res Ther. 2019;15(1):20–5. 10.4103/jcrt.JCRT_30_17 30880749

[28] Piraux E, Reychler G, Vancraeynest D, Geets X, Léonard D, Caty G. High-intensity aerobic interval training and resistance training are feasible in rectal cancer patients undergoing chemoradiotherapy: a feasibility randomized controlled study. Rep Pract Oncol Radiother. 2022;27(2):198–208. 10.5603/RPOR.a2022.0036 36299392 PMC9591035

[29] Mast IH, Gootjes EC, Rütten H, den Hartogh MD, Brouwer CG, Nagtegaal ID, et al. Feasibility and clinical potential of exercise interventions during neoadjuvant chemoradiotherapy in patients with esophageal and rectal cancer. J Sport Health Sci. 2025;14:101060. 10.1016/j.jshs.2025.101060 40419137 PMC12482329

[30] Moug SJ, Mutrie N, Barry SJE, Mackay G, Steele RJC, Boachie C, et al. Prehabilitation is feasible in patients with rectal cancer undergoing neoadjuvant chemoradiotherapy and may minimize physical deterioration: results from the REx trial. Colorectal Dis. 2019;21(5):548–62. 10.1111/codi.14560 30657249

[31] Heldens AFJM, Bongers BC, De Vos-Geelen J, Van Meeteren NLU, Lenssen AF. Feasibility and preliminary effectiveness of a physical exercise training program during neoadjuvant chemoradiotherapy in individual patients with rectal cancer prior to major elective surgery. Eur J Surg Oncol. 2016;42(9):1322–30. 10.1016/j.ejso.2016.03.021 27156145

[32] Brunet J, Price J, Delluc C. An exercise trial for adults undergoing neoadjuvant chemoradiotherapy for rectal cancer proves not feasible: recommendations for future trials. Trials. 2021;22(1):26. 10.1186/s13063-020-04958-z 33407782 PMC7789730

[33] Loughney L, West MA, Moyses H, Bates A, Kemp GJ, Hawkins L, et al. The effects of neoadjuvant chemoradiotherapy and an in-hospital exercise training programme on physical fitness and quality of life in locally advanced rectal cancer patients: a randomised controlled trial (The EMPOWER Trial). Perioper Med. 2021;10(1):23. 10.1186/s13741-021-00190-8 PMC8216760 34154675

[34] Morielli AR, Usmani N, Boulé NG, Severin D, Tankel K, Nijjar T, et al. Exercise during and after neoadjuvant rectal cancer treatment (the EXERT trial): study protocol for a randomized controlled trial. Trials. 2018;19(1):35. 10.1186/s13063-017-2398-1 29329555 PMC5767015

[35] Morielli A, Usmani U, Boulé N, Severin D, Tankel K, Nijjar T, et al. Exercise motivation in rectal cancer patients during and after neoadjuvant chemoradiotherapy. Support Care Cancer. 2016;24(7): 2919–26. 10.1007/s00520-016-3110-9 26847350

[36] Moug SJ, Barry SJE, Maguire S, Johns N, Dolan D, Steele RJC, et al. Does prehabilitation modify muscle mass in patients with rectal cancer undergoing neoadjuvant therapy? A subanalysis from the REx randomised controlled trial. Tech Coloproctol. 2020;24(9):959–64. 10.1007/s10151-020-02262-1 32564236 PMC7429543

[37] Morielli AR, Usmani N, Boul NG, Severin D, Tankel K, Joseph K, et al. Feasibility, safety, and preliminary efficacy of exercise during and after neoadjuvant rectal cancer treatment: a phase II randomized controlled trial. Clin Colorectal Cancer. 2021;20(3):216–26. 10.1016/j.clcc.2021.05.004 34158253

[38] Totton N, Lin J, Julious S, Chowdhury M, Brand A. A review of sample sizes for UK pilot and feasibility studies on the ISRCTN registry from 2013 to 2020. Pilot Feasibility Stud. 2023;9(1):188. 10.1186/s40814-023-01416-w 37990337 PMC10662929

[39] Loughney L, West MA, Kemp GJ, Rossiter HB, Burke SM, Cox T, et al. The effects of neoadjuvant chemoradiotherapy and an in-hospital exercise training programme on physical fitness and quality of life in locally advanced rectal cancer patients (The EMPOWER Trial): study protocol for a randomised controlled trial. Trials. 2016;17(1):24. 10.1186/s13063-015-1149-4 26762365 PMC4710998

[40] Singh B, Hayes SC, Spence RR, Steele ML, Millet GY, Gergele L. Exercise and colorectal cancer: a systematic review and meta-analysis of exercise safety, feasibility and effectiveness. Int J Behav Nutr Phys Act. 2020;17(1):122. 10.1186/s12966-020-01021-7 32972439 PMC7513291

[41] Courneya Kerry S, Vardy Janette L, O’Callaghan Christopher J, Gill S, Friedenreich Christine M, Wong Rebecca KS, et al. Structured exercise after adjuvant chemotherapy for colon cancer. N Engl J Med. 2025;393(1):13–25. 10.1056/NEJMoa2502760 40450658

[42] Loughney L, West MA, Dimitrov BD, Kemp GJ, Grocott MP, Jack S. Physical activity levels in locally advanced rectal cancer patients following neoadjuvant chemoradiotherapy and an exercise training programme before surgery: a pilot study. Perioper Med. 2017;6:3. 10.1186/s13741-017-0058-3 PMC5311720 28228938

[43] Latrille M, Buchs NC, Ris F, Koessler T. Physical activity programmes for patients undergoing neo-adjuvant chemoradiotherapy for rectal cancer: a systematic review and meta-analysis. Medicine. 2021;100(51):e27754. 10.1097/MD.0000000000027754 34941028 PMC8702187

[44] Wallen MP, Hennessy D, Brown S, Evans L, Rawstorn JC, Wong Shee A, et al. High-intensity interval training improves cardiorespiratory fitness in cancer patients and survivors: a meta-analysis. Eur J Cancer Care. 2020;29(4):e13267. 10.1111/ecc.13267 32469144

[45] Zhang X, Wang S, Ji W, Wang H, Zhou K, Jin Z, et al. The effect of prehabilitation on the postoperative outcomes of patients undergoing colorectal surgery: a systematic review and meta-analysis. Front Oncol. 2022;12:958261. 10.3389/fonc.2022.958261 35965591 PMC9372464

[46] Navidi M, Phillips AW, Griffin SM, Duffield KE, Greystoke A, Sumpter K, et al. Cardiopulmonary fitness before and after neoadjuvant chemotherapy in patients with oesophagogastric cancer. Br J Surg. 2018;105(7):900–6. 10.1002/bjs.10802 29601082

[47] Danielsson J, Engström Sid J, Andersson M, Nygren-Bonnier M, Egenvall M, Hagströmer M, et al. Optimizing physical fitness before colorectal cancer surgery (CANOPTIPHYS): the effect of preoperative exercise on pre- and postoperative physical fitness in older people – a randomized controlled trial. J Prim Care Community Health. 2025;16:21501319251346417. 10.1177/21501319251346417 40525350 PMC12174800

[48] Liang J, Zhang X, Xia W, Huang H, Tao J. Promotion of aerobic exercise induce angiogenesis is associated with decline in blood pressure in hypertension result of excavation CHN1. J Am Coll Cardiol. 2020;75(11 Suppl 1):1845. 10.1016/S0735-1097(20)32472-4 33611934

[49] Schaun GZ, Alberton CL, Ribeiro DO, Pinto SS. Acute effects of high-intensity interval training and moderate-intensity continuous training sessions on cardiorespiratory parameters in healthy young men. Eur J Appl Physiol. 2017;117(7):1437–44. 10.1007/s00421-017-3636-7 28488137

[50] Liscu H-D. Short-course radiotherapy versus long-course radio-chemotherapy as neoadjuvant treatment for locally advanced rectal cancer: meta-analysis from a toxicity perspective. Maedica. 2021;16(3):382–8. 34925591 10.26574/maedica.2021.16.3.382 PMC8643566

